# Correction: CTL–doxorubicin (DOX)–gold complex nanoparticles (DOX–AuGCs): from synthesis to enhancement of therapeutic effect on liver cancer model

**DOI:** 10.1039/d2na90075k

**Published:** 2022-10-14

**Authors:** Qiqian Liu, Hui Liu, Pasquale Sacco, Nadia Djaker, Marc Lamy de la Chapelle, Eleonora Marsich, Xiaowu Li, Jolanda Spadavecchia

**Affiliations:** CNRS, UMR 7244, NBD-CSPBAT, Laboratoire de Chimie, Structures et Propriétés de Biomatériaux et d’Agents Thérapeutiques Université Paris 13 Sorbonne Paris Nord Bobigny France jolanda.spadavecchia@univ-paris13.fr; Department of Hepato-biliary Surgery, Shenzhen University General Hospital, Guangdong Provincial Key Laboratory of Regional Immunity and Diseases, Carson International Cancer Shenzhen 518055 China; Department of Life Sciences, University of Trieste Via L. Giorgieri 5 I-34127 Trieste Italy; Department of Medicine, Surgery and Health Sciences, University of Trieste Piazzale Europa 1 I-34127 Trieste Italy; IMMM – UMR 6283 CNRS, Université du Mans Avenue Olivier Messiaen 72085 Le Mans Cedex 9 France

## Abstract

Correction for ‘CTL–doxorubicin (DOX)–gold complex nanoparticles (DOX–AuGCs): from synthesis to enhancement of therapeutic effect on liver cancer model’ by Qiqian Liu *et al.*, *Nanoscale Adv.*, 2020, **2**, 5231–5241. https://doi.org/10.1039/D0NA00758G.

The authors regret that the TEM image and the values from the distribution histogram of nanoparticles were incorrectly shown in Fig. 1A and A1, respectively, appearing in the original article and in the graphical abstract in the online version.

The corrected version of Fig. 1 is shown below:

**Fig. 1:**
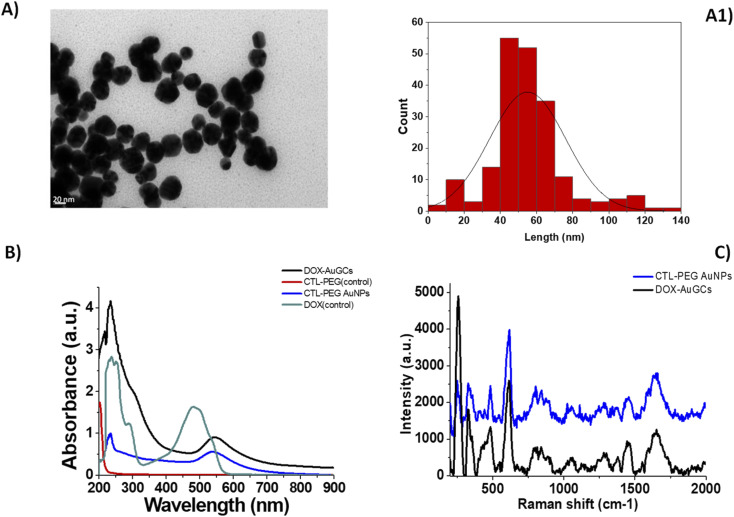
(A) TEM images of DOX–AuGCs and (A1) histogram size nanoparticles (scale bars: 20 nm).

The authors also regret that there was an error in the first sentence below Fig. 1 of the original article. The text originally read: TEM images of DOX–AuGCs display a spherical shape and a narrow dispersion of the nanoparticle size with an average diameter of 50 ± 3 nm (Fig. 1A and A1).

This sentence should read: TEM images of DOX–AuGCs display a spherical shape and a relatively narrow dispersion of the nanoparticle size with an average diameter of 50 ± 30 nm (Fig. 1A and A1).

The Royal Society of Chemistry apologises for these errors and any consequent inconvenience to authors and readers.

## Supplementary Material

